# Double-negative neuromyelitis optica spectrum disorder

**DOI:** 10.1177/13524585231199819

**Published:** 2023-09-23

**Authors:** Yan Wu, Ruth Geraldes, Maciej Juryńczyk, Jacqueline Palace

**Affiliations:** Neurology Department of First Affiliated Hospital of Kunming Medical University, Kunming, China/Nuffield Department of Clinical Neurosciences, Oxford University Hospitals, Oxford, UK; Nuffield Department of Clinical Neurosciences, Oxford University Hospitals, Oxford, UK/Neurology Department, Wexham Park hospital, Frimley Foundation Health Trust, Slough, UK; Department of Neurology, Stroke and Neurological Rehabilitation, Wolski Hospital, Warsaw, Poland; Nuffield Department of Clinical Neurosciences, Oxford University Hospitals, Oxford, UK; J Palace Department Clinical Neurology, John Radcliffe Hospital, Oxford OX3 9DU, UK

**Keywords:** AQP4-IgG, MOG-IgG, double seronegative, neuromyelitis optica spectrum disorder, multiple sclerosis

## Abstract

Most patients with neuromyelitis optica spectrum disorders (NMOSD) test positive for aquaporin-4 antibody (AQP4-IgG) or myelin oligodendrocyte glycoprotein antibodies (MOG-IgG). Those who are negative are termed double-negative (DN) NMOSD and may constitute a diagnostic and therapeutic challenge. DN NMOSD is a syndrome rather than a single disease, ranging from a (postinfectious) monophasic illness to a more chronic syndrome that can be indistinguishable from AQP4-IgG+ NMOSD or develop into other mimics such as multiple sclerosis. Thus, underlying disease mechanisms are likely to be heterogeneous. This topical review aims to (1) reappraise antibody-negative NMOSD definition as it has changed over time with the development of the AQP4 and MOG-IgG assays; (2) outline clinical characteristics and the pathophysiological nature of this rare entity by contrasting its differences and similarities with antibody-positive NMOSD; (3) summarize laboratory characteristics and magnetic resonance imaging findings of DN NMOSD; and (4) discuss the current treatment for DN NMOSD.

## Introduction

Neuromyelitis optica spectrum disorder (NMOSD) is a central nervous system (CNS) autoimmune disease with a predisposition for the optic nerve and spinal cord. The discovery of antibodies to the aquaporin 4 (AQP4) water channel in the majority of patients also highlighted that other CNS locations were often involved.^1^ More recently, with the ability to detect serum myelin oligodendrocyte glycoprotein IgG (MOG-IgG), many AQP4-IgG-negative NMOSD patients have been reported to be MOG-IgG-positive.^2,3^ Although as the majority of those with MOG-IgG have an extended range of clinical phenotypes, this disorder has now been renamed MOG antibody-associated disease (MOGAD). Despite the typical association of NMOSD with either AQP4- or MOG-IgG, there is still a subset of patients who are truly double seronegative and without a diagnostic marker.^2,3^ These patients may sometimes end up with other CNS inflammatory diseases including multiple sclerosis (MS) and sarcoidosis. They may be monophasic (such as para-infectious conditions) or relapsing; thus, double seronegative NMOSD is clearly not a single disease but a syndrome with differing treatment requirements. Indeed, MS drugs may exacerbate antibody-mediated diseases such as AQP4-IgG+ NMOSD or be ineffective in MOGAD^4^, and thus, there is a caution in seronegative NMO.^5^ Thus, the diagnosis can be challenging but has important treatment implications.

**Figure 1.**
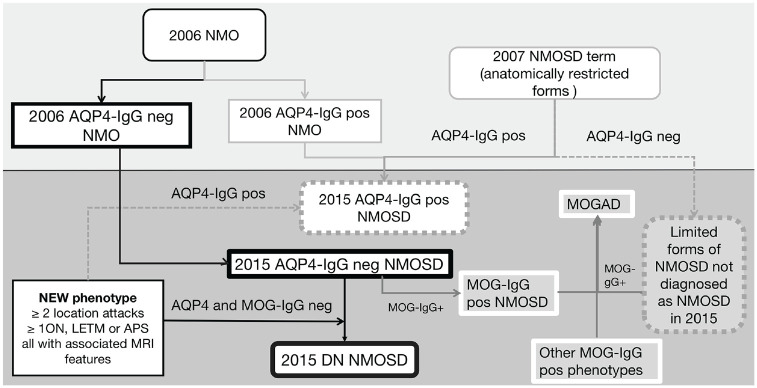
Overview of evolutionary process of NMO/NMOSD diagnostic criteria and the division of antibody-positive diseases and DN NMOSD. APS: area postrema syndrome.

It is worth noting that over time the diagnostic criteria have changed (Figure 1). The term NMO was extended to NMOSD in 2015 and those without AQP4 antibodies were set more rigorous requirements so that those with limited anatomical involvement (such as a long spinal cord lesions or severe bilateral optic neuritis (ON)) previously often referred to as NMOSD^6^ no longer satisfy the 2015 criteria.^1^

In addition, the assays vary in their accuracy across sites and across studies with improved sensitivity and specificities over time. Before assays to conformational MOG-IgG were developed, antibody-negative NMO/NMOSD cohorts were in fact only AQP4-IgG seronegative. Now, antibody-negative disease refers to those negative for both AQP4-IgG and MOG-IgG (DN NMOSD) using the most accurate assay.^1,7^

Thus, interpretation of the data on seronegative NMOSD will need to take into consideration the changing definitions and cohort selection which will depend on the year of the study and the assays used. Herein, we will review the key clinical and laboratory features of seronegative NMOSD, highlighting the differences from AQP4-IgG+ NMOSD, MOGAD, and MS where relevant, and summarize current treatment strategies.

## Epidemiology and demography

The proportion of DN patients in AQP4-IgG-negative NMO/NMOSD patients varies from 0% to 79%,^2,3,8–11^ depending on the cohort selection and assay type used. However, there are likely to be recruitment biases and assay sensitivity issues which overestimate the prevalence as a small epidemiological study recently suggested it is rare with all the local cases being antibody positive.^3^ The median onset age of DN NMO/NMOSD in different studies ranged from 32 (therefore similar to MOGAD) to 43 years (similar to AQP4-IgG+ patients).^3,8,9,12–14^ DN NMO/NMOSD patients have equal sex ratios^12,15^ or a mild female predominance as reported in MOGAD,^8,11,16–19^ and contrasts with the marked female predominance in AQP4-IgG+ patients^8,11,12,15,17^ and the predominance of females in MS. In addition, DN NMO/NMOSD (as with MOGAD patients) reflects the racial background of the population,^9,12,16,19^ and contrasts with the black or Asian predominance in AQP4-IgG+ NMOSD.^20,21^

## Pathophysiology

DN NMOSD is likely heterogeneous; therefore, the pathophysiology might differ between patients, but it may still have some common features (yet undiscovered autoantibodies or complement involvement) as evidenced by shared clinical and radiological features (in particular extensive involvement of the spinal cord during the acute attack) defined in the criteria. Immunology studies in DN NMOSD are few and that they were typically focused on NMOSD in general rather than DN NMOSD specifically. Cerebrospinal fluid (CSF) pleocytosis was reported in a subset (17%–50%) of DN NMOSD with a mean of 3–10 white blood cells^8,13,22^ supporting lymphocytic CNS activation.^9^

Similar to AQP4-IgG+ NMOSD, the inflammatory response in DN NMOSD is increased as reflected by significantly elevated serum proinflammation cytokines. Several studies have shown that the levels of interleukin 6 (IL-6) are high in the CSF of DN NMO/NMOSD patients.^23–25^ In one study, markers of complement activation in the plasma helped differentiate the whole NMOSD group from MS and controls.^26^ In particular, DN NMOSD patients (*n* = 8) had significantly higher terminal complement complex and iC3b when compared with MS patients (*n* = 40).^26^ However, complement activation was not supported by a murine animal model where purified IgG from AQP4-IgG+ patients clearly activated complement, but purified IgG from five DN NMOSD patients did not.^27^ The findings may be reliant on assuming mice will express disease in the same way as humans and that the DN NMOSD patients all have the same pathology which is unlikely.

It remains unclear whether DN NMOSD is primarily an astrocytopathy similar to AQP4-IgG+ NMOSD. Two small cohort studies showed that CSF glial fibrillary acidic protein (GFAP) and glutamine synthetase levels were higher or similar in DN NMOSD when compared with AQP4-IgG+ NMOSD, and clearly higher than in MOGAD and MS, which was supportive of astrocytic injury in DN NMOSD.^22,28^ On the contrary, a recent multicentre study including 17 DN NMOSD patients (all fulfilling 2015 criteria) demonstrated significantly lower CSF GFAP levels in DN NMOSD (and MOGAD) when compared with AQP4-IgG NMOSD.^29^ CSF-S100B indicating astrocyte injury was also noted to be significantly higher in AQP4-IgG+ and DN NMOSD patients than in MOGAD.^22^ CSF neurofilament light chain (NFL) levels were noted to be much higher in DN NMOSD compared with AQP4-IgG+ NMOSD or MOGAD, suggesting that there is neuronal-axonal damage in DN NMOSD patients.^25^

In summary, there is evidence to suggest that neuronal damage in DN NMOSD is as severe as in AQP4-IgG+ NMOSD and more pronounced than in MOGAD or MS. It remains controversial whether DN NMOSD is a primary astrocytopathic disease.

## Clinical phenotypes

The majority of patients labeled as DN NMOSD will have a combination of longitudinally extensive ON, longitudinally extensive transverse myelitis (LETM), and/or NMOSD-typical brain/brainstem attacks because of the 2015 classification (Table 1).^1^ Area postrema attacks are rare, and the features of brain/brainstem attacks are not well characterized (based on our experience may involve various NMOSD-typical regions, including periependymal brainstem, splenium of corpus callosum, and hemispheric subcortical white matter).^1,7,18^ DN NMO/NMOSD cases can be monophasic unlike AQP4-IgG+ NMOSD;^8,12,30^ thus, one would expect a lower frequency of relapsing disease in DN NMO/NMOSD compared with those with AQP4 IgG. The proportion that becomes relapsing will depend on the follow-up time and the referral bias, but figures vary from 24% to 86% (median follow-up 13.1 months to 6.4 years).^8,12,17^ However, of those who relapse in a French DN NMOSD cohort, there were similar mean times to the second attack (3.4 years) when compared with AQP4-IgG+ NMOSD (3.2 years) but shorter times than MOGAD patients (11.3 years).^14^ It is worth noting that AQP-IgG+ NMOSD patients are typically treated with immunosuppressants from the time of diagnosis, which has been shown to enhance the interval between relapses and decrease the frequency of subsequent relapses. Reports also vary as to whether relapse frequency is less or more in DN NMO/NMOSD than in MOGAD.^8,9,31^

**Table 1.** Comparisons of clinical features and paraclinical tests between DN NMOSD, AQP4-IgG+ NMOSD, MOGAD, and MS.

	DN NMOSD	AQP4-IgG+ NMOSD	MOGAD	RRMS	References
Median onset age (year)	32–43	37–45	27–38		3,8,9,12–14
Sex predilection	Equal sex ratios or mild female predominance	Marked female predominance	Mild female predominance	Female predominance	8,11,12,15–19
Race	Background population	Black or Asian predominance	Background population	Any ethnic background but most common in white	9,12,16,19
Monophasic course	++	−	++	−	8,12,30
**Clinical phenotypes**					
Bilateral ON	+	++	++		9,18
Simultaneous or sequential occurrence of ON and TM	++	+	++	−	9,12,13,15
TM	++	++	+	++	22
Disability	Severe	Severe	Moderate-mild	Good recovery from relapses	8,12–15,17,22,31
Severe visual loss	++	++	+		8,17,18
**CSF findings**					
Pleocytosis	↑	↑	↑↑	—	8,13,22
Lymphocytosis	+	+	+		9
OCBs	±	±	±	+	8,14
GFAP	↑	↑↑↑	↑↑	↑	22,28,29
NFL	↑↑↑	↑↑	↑	↑	25
IL-6	↑	↑↑	↑(↑↑)	—	23–25
**Image biomarkers**					
**Brain lesions**	++	++	+	+++	8,18
Medullary lesions	+	++	+		8
**Spinal cord**					
LETM	Most cervicothoracic	Most cervical	Most thoracolumbar		8
Regions of the cord	Most dorsal and more central portion	More central portion	Any region, predilection to conus	Eccentric	8,18

() Brackets are data from direct comparison literature for AQP4-IgG+ NMOSD and MOGAD of larger numbers whose data are different from studies with the whole three groups. CSF: cerebrospinal fluid; DN NMOSD: double-negative neuromyelitis optica spectrum disorders; GFAP: glial fibrillary acidic protein; IL-6: interleukin 6; LETM: longitudinally extensive transverse myelitis; MOGAD: myelin oligodendrocyte glycoprotein antibody-associated disease; NFL: neurofilament light chain; OCBs: oligoclonal band; ON: optic neuritis; RRMS: relapsing-remitting multiple sclerosis; TM: transverse myelitis.

Although the criteria^1^ set out to make DN NMO/NMOSD patients similar to AQP4-IgG+ patients, DN NMO/NMOSD patients have more frequent occurrence of simultaneous ON and LETM (likely because they are required to have two anatomical locations involved) but less than those with MOG-IgG.^12,14,18^ When compared with MOGAD, they are more likely to have LETM, more likely in the cervical spine, and less likely to have bilateral simultaneous ON or brain lesions.^8,9,12,13–15,17,18,22^

Attacks in patients with DN NMO/NMOSD cause similar or worse residual disability when compared with AQP4-IgG+ NMOSD and, therefore, much worse than in MOGAD despite similar severity at nadir.^8,12–15,17,22,32^ Twenty-fifty percent of DN NMOSD patients have been reported to be left with severe visual impairment.^8,17,18^ Older onset patients have worse outcomes than young onset patients.^15^ It should, however, be noted that milder forms of DN NMOSD exist as reported in a recent study aimed at unsupervised subgrouping of antibody-negative patients with NMOSD features based on clinical characteristics and conventional and nonconventional imaging where one of the identified subgroups characterized by co-occurrence of LETM and ON was associated with a good clinical recovery.^33^

## Double antibody-negative patients with NMOSD features who do not fulfill the current diagnostic criteria

DN patients with limited forms of NMOSD including isolated LETM and isolated ON with poor recovery are a major diagnostic and therapeutic challenge in neuroimmunology clinics. Those with LETM might be difficult to differentiate from MS with coalescing spinal cord lesions and few or no brain lesions. Consideration of interval from symptom onset to scan (LETM fragmentation) and careful scrutiny of axial cord images (wedge-shaped peripheral lesions in MS versus central or holocord lesion in NMOSD) are essential. Inclusion of sequences allowing for a better assessment of cortical lesions, for example, double inversion recovery, might prove helpful in the future.^33^ DN patients with overlapping features of NMOSD and MS have been largely understudied as they might not fulfill neither MS nor NMOSD criteria and, therefore, are not included in research cohorts (“forgotten patients”). In recent studies aimed at elucidating the structure of DN NMOSD cohort,^33,34^ the inclusion criteria were broadened to include all patients who have at least one NMOSD feature.

## Laboratory characteristics

The presence of coexistent autoimmunity is more commonly observed in NMOSD compared with MS and MOGAD, and this particularly holds true for AQP4-IgG NMOSD which has a well-recognized overlap with systemic lupus erythematosus, Sjogren’s disease, autoimmune thyroid disorders, and myasthenia gravis (Table 1).^35,36^ Compared with around half of AQP4-IgG+ NMOSD, 4.6%–48% DN NMOSD patients were tested positive for antinuclear antibodies.^8,13^ Unmatched CSF oligoclonal bands (OCBs) were found in 8% to 23% of DN NMOSD,^8,14^ which is lower than MS.

## Imaging biomarkers

**Figure 2.**
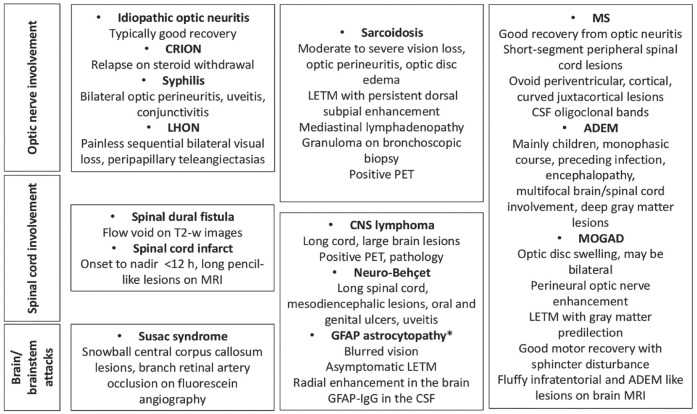
Differential diagnoses in patients with relapsing/progressive atypical DN-NMOSD.

As the criteria dictate DN NMOSD imaging is predominantly characterized by longitudinally extensive lesions in the cord (continuous involvement of at least three segments) and brain imaging not suggestive of MS (therefore not showing ovoid lesions perpendicular to lateral ventricles, inferior temporal lobe lesions or curved juxtacortical lesions) (Table 1). It is noteworthy that there are many causes of LETM and that differential diagnosis is essential in particular in DN patients (Figure 2).^37^

## Brain lesions

In the inebilizumab clinical trial, 4 out of 17 (23.5%) AQP4-IgG-negative NMOSD patients had a history of brain or brainstem lesions at baseline.^38^ Brain imaging features reported in seronegative NMOSD patients include extensive hemispheric white matter lesions,^5^ diffuse splenium lesions,^5,39^ and lesions adjacent to fourth ventricle.^39^

An Oxford study identified brain imaging discriminators via discriminant analysis of principal components combining the magnetic resonance imaging (MRI) features of MOGAD and AQP4-IgG+ NMOSD to differentiate from MS.^40^ The use of such a discriminatory model may be helpful in subclassifying patients with DN NMOSD/MS overlapping syndromes^33,39^ who in clinical practice are one of the main diagnostic challenges. A deep-learning algorithm trained with brain MRI scans of AQP4-IgG+ NMOSD and MS patients was also validated to be able to forecast whether seronegative NMOSD-like manifestations would be developed into antibody-positive NMOSD or MS or stay DN NMOSD.^41^

## Spinal cord

**Figure 3.**
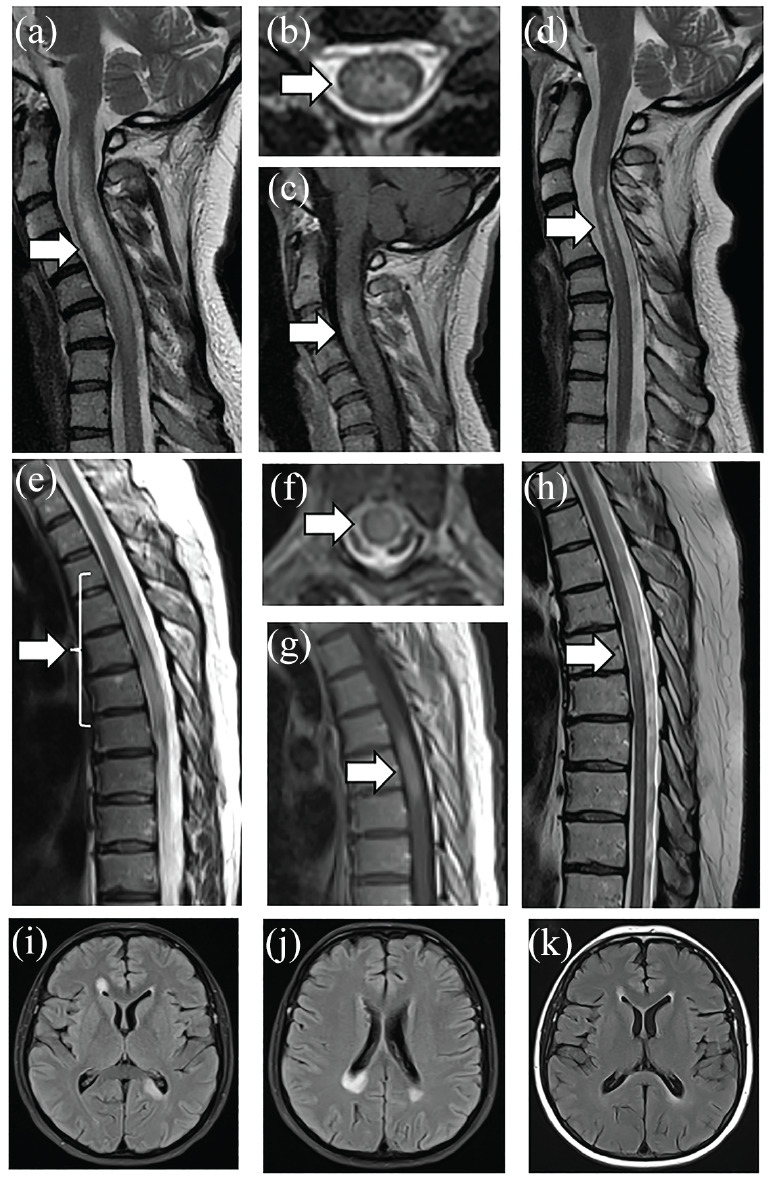
(a to d) A 41-year-old women ultimately diagnosed with AQP4-IgG NMOSD presented with neck pain and left arm weakness with rapid progression more than 2 weeks to quadriplegia and breathing difficulties. Cervical MRI showed longitudinally extensive lesion on T2-weighted imaging with cord swelling (a), central location on axial images (b), and ring-like contrast enhancement (c). The patient was treated with intravenous methylprednisolone followed by plasma exchange with improvement. Follow-up imaging 9 months later showed residual T2-weighted hyperintensity in the central portion of the cord (d). (e to k) A 45-year-old women presented with numbness and weakness in the left leg progressing more than 3 weeks to severe paraplegia with double incontinence and Th6 sensory level despite intravenous methylprednisolone. Spinal cord MRI showed a longitudinally extensive lesion spanning the cord continuously from Th3 to Th7 with mild cord swelling (e). The lesion was located centrally (f) and partially contrast-enhancing (g). Brain MRI showed large poorly demarcated periventricular lesions located in the corpus callosum (i, j). Patient’s serum was negative for both AQP4- and MOG-IgG on fixed cell-based assays locally. The patient was treated with plasma exchange with partial recovery but remained wheelchair-bound. Follow-up imaging of the spinal cord performed 9 months after the onset of symptoms showed partial resolution of LETM lesion (h) and absence of contrast enhancement (not shown). Follow-up brain MRI showed almost complete resolution of brain lesions and absence of new lesions (k). Repeat antibody testing, including live cell-based assays performed in Oxford, remained negative for both AQP4 and MOG antibodies. The patient was diagnosed with seronegative NMOSD and started on rituximab. Both patients consented for their images and clinical summaries to be included in the article (Department of Neurology, Stroke and Neurological Rehabilitation, Wolski Hospital, Warsaw, Poland).

As the criteria require MRI typical changes for all attacks in AQP4-IgG-negative NMOSD patients but not for those with AQP4-IgG, LETM is a prerequisite for a DN NMOSD TM attack. Examples of AQP4-IgG and double seronegative LETM in the acute phase and follow-up are shown in Figure 3. In general, DN NMOSD LETM lesions tend to involve the cervical or thoracic cord (or both) rather than conus (more typical of MOGAD).^8^ In addition, LETM lesions in DN NMOSD (4.5 vertebral segments) are on average shorter than in antibody-positive (both AQP4-IgG+ NMOSD and MOGAD) and similarly to antibody-positive patients axially tend to involve the central cord (79.1%).^8^ Of note, short TM lesions may occur in AQP4-IgG+ NMOSD and MOGAD.^42,43^ It remains to be elucidated whether bright spotty lesions considered to be a highly specific biomarker of AQP4-IgG NMOSD-associated transverse myelitis also occur in DN NMOSD.^44^

## Combined clinical imaging and metabolic methods to differentiate DN NMOSD

As DN NMOSD has no diagnostic biomarker and is a heterogeneous group, combined methods may provide more information. With the application of NMO/MS discriminators by using principal components combining clinical, laboratory, and MRI features, relapsing DN NMOSD patients have been shown to divide into four different patterns: MS-like (often had central vein sign and cortical lesions); spinal MS-like (had short-segment myelitis and no MS-like brain lesions); classic NMO-like (high percentage of bilateral ON and LETM with normal brain appearance; and NMO-like with brain involvement (a history of NMOSD-like brain lesions and LETM),^33^ indicating a high heterogeneity and complexity of DN NMOSD. Interestingly, identified subgroups differed significantly with respect to N-acetylaspartate a neuronal-axonal marker in the cervical spinal cord with lower N-acetylaspartate in “MS-like” patients along with a lower occurrence of LETM compared with “classic NMOSD-like” patients.^34^ The metabolic differentiators measured in the patient serum: myoinositol and formate, previously shown to differentiate MS from the two antibody conditions together, were also shown to validate principal component analysis spontaneously separating DN NMOSD-like groups into “MS like” and “NMOSD like” groups.^45^

## Current treatment

There is limited evidence to guide treatment in DN NMOSD, and most experts treat acute attacks similarly to that for antibody-positive patients and immune-suppress relapsing patients with the conventional therapies used for AQP4-IgG+ NMOSD and MOGAD disease. Studies suggested that during relapse DN NMO/NMOSD patients were less likely to be as corticosteroid responsive and less likely to relapse with corticosteroid cessation than MOGAD patients.^9,32^

At present, no relapse preventive agents are approved for DN NMOSD and so conventional immunosuppressants are often empirically used. Because several MS DMTs are ineffective or exacerbate relapses in AQP4-IgG+ NMOSD patients (such as interferon-β, glatiramer acetate, natalizumab, alemtuzumab, and fingolimod)^46^ and because a subgroup of DN NMOSD patients may have below assay threshold levels of AQP4 IgG or have other undiscovered antibodies, such treatments are also avoided. In those with MS/NMOSD overlapping syndromes, anti-B-cell therapies may be alternative treatments because these are suitable for antibody diseases and MS. In a multicentre retrospective analysis of NMOSD patients, in those who were seronegative, annualized relapse rate declined from 1.93 to 0.12 following rituximab treatment.^47^ First-line therapy with glucocorticoids, azathioprine, and mycophenolate mofetil, also rituximab, methotrexate, and mitoxantrone may be used.^10,47,48^ Comparative analyses of the effect of all the immunosuppressants on relapse rate did not reveal significant differences among AQP4-IgG+, MOGAD, and DN groups.^17^ One retrospective study including eight AQP4-IgG-negative NMOSD patients reported an 86% reduction in annual relapse rate after tacrolimus treatment given for a median of approximately 4 years with a 62.5% relapse-free rate over this period. MOG-IgG status was not tested to know the effect in true DN NMOSD.^49^ In patients with relapsing DN LETM, who would have fulfilled the 2007 NMOSD criteria,^6^ early immune suppression after the onset attack reduced the risk of a second relapse from 32% to 9% at 18 months.^50^

Tocilizumab (TCZ, an anti-IL-6 receptor monoclonal antibody (mAb)) showed some promise in a couple of studies; however, they were either small and only subanalyzed AQP4-IgG-negative NMOSD rather than DN NMOSD patients Tocilizumab versus azathioprine in highly relapsing neuromyelitis optica spectrum disorders: to GO (TANGO)^51^ or were small and retrospective not excluding regression toward the mean.^52^ Two further small TCZ studies in DN NMOSD patients suggested some effect.^53,54^ Despite some promise from nonrandomized reports and studies of anti-IL-6 receptor mAb treatments, two randomized controlled trials (RCTs) of satralizumab, also an anti-IL-6 receptor mAb effective in AQP4-IgG+ NMOSD, showed no efficacy in those without AQP4 IgG.^55,56^ The role of IL-6 in DN NMO/NMOSD is thus unclear as it has been reported to be increased in the CSF of some DN patients although less than in those with AQP4 antibodies.^23,25^ It may be that a subset of DN NMOSD patients might respond to anti-IL-6R drugs.

## Newer licensed NMOSD drugs

Very few DN NMOSD patients were enrolled in the inebilizumab study (an anti-CD19 drug targeting B cells). Although the study failed to show a therapeutic effect in DN NMOSD patients, no relapses occurred after the second dose and patients remained relapse-free at nearly 6.5 months of randomized controlled period.^38,57^ The eculizumab study did not recruit AQP4-IgG-negative patients because it was felt it was most likely that only AQP4 IgG-positive patients would have a complement-mediated pathology.^58^

## Challenges and opportunities for future research

DN NMOSD is heterogeneous and has no specific diagnostic marker. Differential diagnosis with MS in particular, but also other mimicking conditions, including sarcoidosis, is essential. Further characterizing patients into monophasic and relapsing subgroups, separating out those with MS-like features and other mimics using unsupervised analysis and biomarkers, will produce more homogeneous cohorts to study the pathogenesis and treatment response.

**Take-home messages**

1. DN NMOSD is a syndrome, whose definition has changed over time, and the criteria are more rigorous than those for AQP4-IgG+ NMOSD.

2. Accurate diagnosis of DN NMOSD requires high-sensitivity live cell-based assays for both MOG-IgG and AQP4-IgG since antibody-positive patients might be misdiagnosed as DN NMOSD with less accurate assays. Consideration of NMO mimics in those with relapsing disease is important (Figure 2).

3. Although in contrast to AQP4-NMOSD, DN NMOSD may be monophasic or relapsing, it can lead to as severe disability as AQP4-NMOSD.

4. Conventional immunosuppressants reduce relapse risk as suggested by available data but good RCT is lacking.
